# Inhaled Dry Powder of Antiviral Agents: A Promising Approach to Treating Respiratory Viral Pathogens

**DOI:** 10.3390/v17020252

**Published:** 2025-02-12

**Authors:** Tushar Saha, Zia Uddin Masum, Anik Biswas, Moushumi Afroza Mou, Sohag Ahmed, Tamal Saha

**Affiliations:** 1 Mastaplex Ltd., Centre for Innovation, University of Otago, Dunedin 9016, New Zealand; 2 College of Pharmacy and Health Sciences, St. John’s University, Queens, New York, NY 11439, USA; zia.masum22@my.stjohns.edu; 3 Materials and Nanotechnology, North Dakota State University, Fargo, ND 58105, USA; anik.biswas@ndsu.edu; 4 Department of Biological Science, St. John’s University, Queens, New York, NY 11439, USA; moushami.mou22@my.stjohns.edu; 5 Department of Chemistry, West Virginia University, Morgantown, WV 26506, USA; sa00123@mix.wvu.edu; 6 International Centre for Diarrheal Disease Research, Bangladesh, Dhaka 1212, Bangladesh; tamal.saha@icddrb.org

**Keywords:** dry powder, antiviral, respiratory, pathogens, infections

## Abstract

Inhaled dry powder formulations of antiviral agents represent a novel and potentially transformative approach to managing respiratory viral infections. Traditional antiviral therapies in the form of tablets or capsules often face limitations in terms of therapeutic activity, systemic side effects, and delayed onset of action. Dry powder inhalers (DPIs) provide a targeted delivery system, ensuring the direct administration of antivirals to the infection site, the respiratory tract, which potentially enhance therapeutic efficacy and minimize systemic exposure. This review explores the current state of inhaled dry powder antiviral agents, their advantages over traditional routes, and specific formulations under development. We discuss the benefits of targeted delivery, such as improved drug deposition in the lungs and reduced side effects, alongside considerations related to the formulation preparation. In addition, we summarize the developed (published and marketed) inhaled dry powders of antiviral agents.

## 1. Introduction

Respiratory viral infections (RVIs) have a remarkable impact on global health, which can be easily observed through the outbreaks of different epidemics and pandemics [1]. In addition, RVIs affect a large number of patients each year [2]. RVIs can be either contagious or non-contagious. Vaccines are the preferred treatment approach in combating contagious RVIs; however, their development and distribution is a long, complex process [3]. This can be further delayed or failed through antigenic drift resulting from mutations [4]. Again, vaccine effectiveness may diminish over time and varies among different variants of the respective RVIs [5,6]. In addition to this, many people have vaccine hesitancy, which was observed during the COVID-19 pandemic [7,8]. So, besides vaccines, small molecules can play a significant role as a main or adjunct therapy. Small molecules can be the principal treatment approach for non-contagious RVIs. For the effective treatment of any disease, delivering rightly chosen drugs through an appropriate route is a prerequisite [9]. Suboptimal drug concentrations at the site of infection due to improper dosing and route can lead to less effective outcomes and drug resistance [10]. The inhalation delivery of drugs has been a significant route of administration for the treatment of respiratory disorders [11,12]. Since RVIs primarily affect the respiratory tract, delivering antivirals directly to the infection site is the most logical approach, ensuring effective drug concentration with a low dose, minimal side effects, and bypassing first-pass metabolism. Studies on animal models have also showed that the inhaled delivery of drugs is more effective compared to conventional oral or injectable dosage forms for respiratory diseases [13]. However, it is crucial to use the appropriate device to effectively deliver these drugs to the site of infection for optimal therapeutic effect. Numerous devices have been developed over time to deliver drugs to the respiratory tract/lung. The widely used devices for inhalation are dry powder inhalers (DPIs), pressurized metered-dose inhalers (pMDIs), and nebulizers [14]. Each device has its own set of advantages and disadvantages. The key features of these delivery devices are mentioned in Table 1. The selection of the device depends on various factors like dosage requirements, ease of use, product stability, and safety considerations [15]. Usually, a high dose is required to treat RVIs [16]. Researchers have reported different thresholds for addressing a high dosage in terms of powder inhalation, which are ≥5 mg, >2 mg, and >1 mg [17,18]. However, it is important to realize that these high doses for inhalation are much lower than those used in oral dosage forms. If we compare the existing delivery devices for inhalation, delivering a high dose of medication into the lungs using a metered dose inhaler can be challenging. The delivery efficiency of pMDIs is not satisfactory. DPIs and nebulizers can both deliver high doses of antimicrobial agents [19]. However, nebulizers have certain limitations, including a high administration time, poor delivery efficiency, and special set up requirements [19]. Additionally, nebulizer formulations are mainly liquid-based, for which they are less stable. Moreover, the treatment of contagious respiratory pathogens using nebulizers requires specific facilities. In contrast, DPIs offer greater stability and can effectively deliver poor water-soluble drugs. Furthermore, the risk of viral transmission is higher with nebulizers but lower with DPIs [19]. These advantages make DPIs an ideal dosage form for the treatment of RVIs. In this review article, we discuss the factors affecting a successful DPI formulation and currently developed and marketed DPIs for RVIs. Then, we discuss the untapped combinations of inhaled dry powder that needs to be explored by researchers. This review provides both academics and the pharmaceutical industry with an updated overview of the developed inhalable dry powders of antiviral agents, which will be required to combat different respiratory viral pathogens efficiently.

## 2. A Brief Description of Common Respiratory Viral Pathogens

Respiratory viral pathogens are a diverse group of viruses responsible for infections in the respiratory tract, ranging from mild illnesses to severe diseases. These viruses spread primarily through respiratory droplets, direct contact, and occasionally via aerosols, contributing significantly to morbidity and mortality worldwide. They target the respiratory epithelium, leading to inflammation and a cascade of immune responses, which vary based on the virus and the host’s immune status. The following sections provide a brief overview of the most prevalent respiratory viral pathogens.

### 2.1. Influenza Virus

Influenza viruses, belonging to the Orthomyxoviridae family, are enveloped viruses with segmented, negative-sense single-stranded RNA genomes. Among the four genera of this family—types A, B, C, and D—only types A and B are clinically significant in humans [20]. Influenza A, in particular, is a highly pathogenic virus, recognized as a leading viral cause of death in the industrialized world, accounting for approximately 20,000 deaths annually in the United States [21,22]. The pathogenicity and cell tropism of influenza viruses are partly determined by the cleavability of the hemagglutinin (HA) protein by host enzymes [23]. HA initiates infection by binding to receptors on host cells. The virus also undergoes antigenic shift and drift, allowing it to evade host immunity [24]. Influenza is a major public health concern, particularly in the Northern Hemisphere, where it typically occurs between December and April [25]. High-risk groups include older adults, young children, and individuals with chronic diseases affecting the renal, cardiac, and respiratory systems. Symptoms of influenza include the sudden onset of fever, chills, muscle aches, headaches, fatigue, cough, and generalized weakness, which can last up to two weeks. Complications from influenza can include secondary bacterial pneumonia, post-influenza encephalitis, cardiac arrhythmias, and secondary bacterial infections such as Staphylococcus aureus-induced myositis [26,27].

### 2.2. Respiratory Syncytial Virus

Respiratory syncytial virus (RSV) is part of the Paramyxoviridae family, under the Pneumoviridae subfamily [28]. It has a lipid envelope and a single-stranded negative RNA genome. As its name suggests, RSV primarily replicates in the ciliated epithelial cells of the respiratory tract [29]. RSV is categorized into two subgroups, A and B, which are differentiated based on how their two major surface proteins respond to monoclonal antibodies. The G protein aids in viral attachment to host airway-ciliated cells, while the F protein facilitates fusion between the virion and host cell [30]. Once fusion occurs, the virion releases its nucleocapsid into the host cytoplasm, allowing the RNA to enter the cell. The M protein coordinates the assembly of envelope proteins with nucleocapsid proteins (N, P, and M2-1) and assists in budding immature virions from the host cell membrane [31,32]. RSV continues to be a widespread and recurring infectious disease. Infants are at the highest risk of severe RSV infection [33], while older adults are the second-highest risk group, contributing significantly to the RSV disease burden [34]. In most individuals, RSV manifests as common cold-like symptoms, but in vulnerable children, it can progress to bronchiolitis or pneumonia, leading to increased morbidity or even mortality [35]. In adults, RSV infections are typically re-infections that range from mild to moderate in severity. However, certain high-risk populations, such as frail elderly individuals living at home or in long-term care facilities, those with chronic pulmonary diseases, and the severely immunocompromised, are at risk for severe disease [36].

### 2.3. SARS-CoV-2

Coronaviruses (CoVs) have triggered three major outbreaks in the last 20 years: severe acute respiratory syndrome (SARS), Middle Eastern respiratory syndrome (MERS), and, more recently, COVID-19 [37]. Since 2019, approximately 35 million people have been infected with SARS-CoV-2, resulting in over 1 million deaths across 235 countries, regions, or territories [38]. CoVs, part of the Coronaviridae family, are enveloped viruses with positive-sense single-stranded RNA genomes, classified under the Betacoronavirus genus, group 2 [39]. The spike protein facilitates binding to the ACE2 receptor, and four structural proteins are involved in the virus’s entry into host cells. Once inside, the viral RNA is reorganized and replicated in the endoplasmic reticulum [40,41]. The assembly of mature SARS-CoV-2 virions occurs in the ER-Golgi intermediate compartment (ERGIC), with recent evidence indicating that newly formed virions are transported to the cell surface via lysosome trafficking [42,43]. Infected adults most commonly experience fever and cough [44]. Children, when symptomatic, tend to exhibit fewer symptoms than adults [45]. Over 100 symptoms have been reported in individuals with post-COVID conditions, with the most common including fatigue, memory issues, shortness of breath, depression, anxiety, loss of smell, sleep disturbances, and joint pain. Tachycardia is a less frequently observed symptom [46,47].

### 2.4. Rhinovirus

Human rhinoviruses (HRVs) are the primary cause of the common cold and are the second most common virus involved in bronchiolitis and pneumonia in children. HRV-induced bronchiolitis is linked to a higher risk of recurrent wheezing or asthma [48,49]. HRV belongs to the Picornaviridae family, which includes nine genera, six of which are pathogenic to humans: enterovirus, rhinovirus, hepatovirus, parechovirus, cardiovirus, and kobuvirus. HRV is further divided into three species: HRV-A, HRV-B, and HRV-C [50]. These non-enveloped viruses are approximately 30 nm in size, with a positive single-stranded RNA (ssRNA) genome of 7200 base pairs. The genome consists of a single gene encoding 11 proteins, including four capsid proteins (VP1, VP2, VP3, and VP4) that protect the ssRNA genome. VP1, VP2, and VP3 contribute to the virus’s antigenic diversity, while VP4 helps anchor the ssRNA to the capsid [51]. HRVs utilize the ICAM-1, LDLR, or CDHR3 receptors for entry into host cells. Structural proteins VP1 and VP3 interact with VP0 precursors to form protomers, which then assemble into pentamers and complete the capsid. The VP0 precursor remains unprocessed until virus assembly, and the final autocatalytic cleavage, known as “maturation cleavage”, occurs during this process. Progeny viruses are released through the lysis of the host cell’s plasma membrane [52,53]. Clinical presentations of rhinovirus infection in otherwise healthy individuals can range from asymptomatic cases to the common cold, wheezing, and pneumonia. Viral-bacterial interactions are often implicated in rhinovirus-associated conditions such as otitis media, sinusitis, and pneumonia. In immunocompromised individuals, rhinoviral infections can become chronic or life-threatening [54,55].

### 2.5. Adenovirus

Adenoviruses (AdVs) are non-enveloped, double-stranded DNA viruses that usually cause mild infections affecting the upper or lower respiratory tract, gastrointestinal (GI) system, or conjunctiva [56]. At present, around 110 types and genotypes of human adenoviruses (HAdVs) have been identified and are classified into seven species (A–G) [57]. AdVs utilize various receptors, attachment factors, and facilitators to aid in entry and infection, with receptors directly binding to the virion [58]. For example, AdV-C enters cells through dynamin-dependent endocytosis after receptor binding, while AdV-B can enter either through dynamin-dependent or independent endocytosis and penetrates into the cytosol from nonacidic early endosomes [59]. To access the cytoplasm, AdVs employ the amphipathic helix of the internal protein VI to break the endosomal membrane [60]. The virions stay in early endosomes for about 5–10 min before crossing the membrane, though there is significant variability between cells [61]. The viral DNA then separates from the capsid at the nuclear pore complex (NPC), releasing double-stranded DNA associated with hundreds of VII molecules. These VII molecules are critical for protecting the viral DNA from double-stranded break repair mechanisms [61,62].

Adenoviruses have a high transmission rate and are a common cause of respiratory illness in young children [63]. Additionally, extrapulmonary manifestations of adenovirus infections have been reported, including nephritis, cystitis, meningoencephalitis, myocarditis, coagulopathies, and gastroenteritis [64,65,66]. Among immunocompromised patients, 80% experienced severe systemic symptoms such as malaise, lethargy, fatigue, night sweats, gastrointestinal symptoms, and respiratory complaints [67].

## 3. Considering Factors for a Successful Dry Powder Formulation Development

The successful development of a DPI depends on multiple factors and requires meticulous planning. As previously mentioned, respiratory viral infections (RVIs) often necessitate high doses of medication. Consequently, stable and highly aerosolizable dry powder formulations capable of delivering these high doses are crucial for effective treatment. Various methodologies, including milling, crystallization, spray-drying, and spray-freeze drying, have been explored for the development of DPIs [68,69]

Milling involves the physical breakdown of larger particles into smaller ones. Two widely used methods are ball milling and jet milling. In ball milling, particles are broken down mechanically through the motion of one or more balls, a process that can occur with or without a liquid medium. Jet milling, on the other hand, accelerates particles at high velocities, causing collisions that reduce particle size [70,71]

Crystallization focuses on forming crystals, primarily from a solution. This process can be achieved through techniques such as cooling, evaporation, precipitation, or their combinations. The method is chosen based on the solute’s properties and the desired crystal characteristics [72].

Spray-drying is a one-step process in which a liquid feed is atomized into fine droplets and dried rapidly at high temperatures. The feed can be a solution, suspension, or emulsion. Common solvents used in spray-drying include acetone, chloroform, ethanol, methanol, acetonitrile, and water. Solvent residues must adhere to the International Council for Harmonization (ICH) guidelines. The resulting dry powder is collected from a cyclone separator, and spray-drying can operate in open or closed modes. Open mode, using air as the drying gas, is suitable for aqueous solvents, while closed mode, using nitrogen, is employed for organic solvents [73,74].

Spray–freeze-drying combines elements of spray-drying and freeze-drying to produce dry powders. The drug solution is atomized into small droplets, rapidly frozen using cryogenic fluids like liquid nitrogen, and subsequently lyophilized to remove ice and water. This process is particularly useful for heat-sensitive compounds [75].

Emerging methods such as thin-film freezing (TFF) offer promising advancements. In TFF, a drug and stabilizer solution is rapidly frozen on a cryogenically cooled surface, and the solvent is removed via sublimation in a freeze-dryer. This method produces powders with enhanced dispersibility and stability [76].

**Figure 1:**
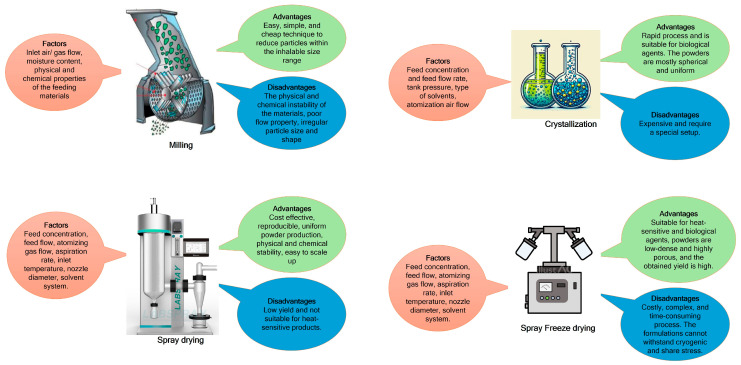
Advantages, disadvantages, and key factors of different preparation techniques of inhalable dry powders.

Each of these techniques has its own advantages and limitations (Figure 1). Process parameters must be carefully optimized to ensure successful DPI development. The selection of a suitable technique depends on the physicochemical properties of the drug and its intended pulmonary delivery application.

**Figure 2:**
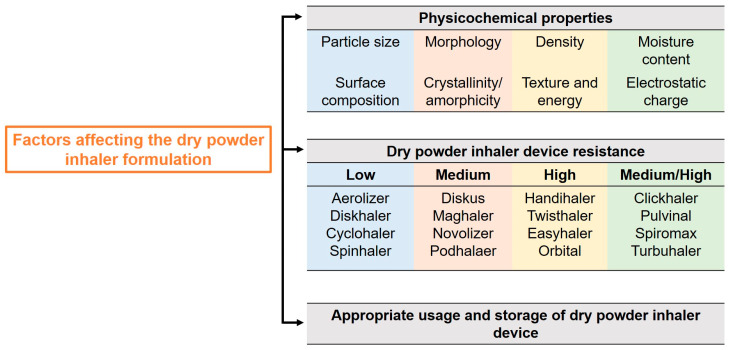
Factors affecting the dry powder inhaler formulation.

The development techniques of DPIs may vary, but the formulation challenges and critical considerations remain consistent. Factors such as formulation characteristics, device design, and proper usage and storage must be taken into account (Figure 2). The primary objective of a DPI for RVIs is to deliver the maximum amount of antiviral agent(s) to the respiratory tract, particularly the deep lung region. This requires high aerosolization efficiency, which is influenced by several physicochemical properties, including particle size, shape, density, surface energy, moisture content, and particle interactions [77,78].

For effective deposition in the small airways and alveolar regions, particles must have an aerodynamic diameter (d_ae_) of 1–5 µm [79]. Particles larger than 5 µm typically settle in the upper airways, while those smaller than 1 µm are often exhaled [79]. The aerodynamic diameter depends on the particle size, density, and morphology, as shown in Equation (1) [79]:

d_ae_ = dv √(ρ/X) (1)

where d_ae_ is the aerodynamic diameter, dv is the particle diameter, ρ is the particle density, and X is the dynamic shape factor.

The aerodynamic diameter increases with particle size if the density and shape factor remain constant. Studies have shown that porous particles with low density and a physical diameter exceeding 5 µm can efficiently reach the deep lung [80]. In terms of morphology, particles resembling pollen with petal-like surfaces demonstrate better deposition potential than those of the same aerodynamic size but different shapes [81]. Additionally, flake- and wrinkle-shaped particles undergo greater deagglomeration, enhancing aerosolization, while particles with low contact areas show reduced agglomeration [81].

Surface energy significantly impacts aerosolization properties, with lower surface energy enhancing aerosolization and higher surface energy promoting agglomeration [82]. Factors such as humidity, moisture, crystallinity, surface texture, and functional groups influence surface energy [82]. For instance, crystalline powders typically aerosolize better than amorphous powders due to their lower surface energy, which reduces particle interaction. However, higher moisture content in the formulation impairs aerosolization and affects drug deposition in the lungs [82]. Hygroscopic materials absorb environmental moisture, altering surface energy and particle interactions, which in turn affect flow properties.

Particle interactions, both cohesive and adhesive, also play a vital role in aerosolization [83]. These interactions are particularly critical for small particles (<10 µm), where gravitational forces are negligible. Various forces, including van der Waals, electrostatic, capillary, and mechanical interlocking, as well as particle morphology and characteristics, influence these interactions [83]. Higher electrostatic charges and van der Waals forces reduce aerosolization efficiency, causing deposition in the upper lungs. Capillary forces, more prominent in hydrophilic materials, further decrease aerosolization by forming solid bridges. Irregularly shaped particles with rough surfaces exhibit higher interactions due to mechanical interlocking, whereas smooth surface particles demonstrate reduced interactions [84].

Particle surface properties significantly influence aerosolization efficiency by affecting interparticle interactions, dispersion, and deposition. High surface energy and electrostatic forces can lead to particle aggregation, reducing aerosolization, while surface roughness and hydrophobicity enhance dispersibility.

The effectiveness of dry powder aerosolization also depends on selecting appropriate DPI devices [85]. Since DPIs are patient-driven, their resistance levels (low, medium, high) can impact the inspiratory flow rate (IFR) and, consequently, drug deposition in the lungs. For example, studies on healthy volunteers show lung deposition percentages for different commercial DPIs as follows: Novolizer (20–32%), Easyhaler (19%), Diskhaler (12%), and Turbuhaler (15–25%). However, achieving optimal flow rates may be challenging for patients with compromised lung function, necessitating careful consideration of these factors during DPI development.

The proper use and storage of DPIs are essential for achieving maximum therapeutic outcomes. Incorrect usage, such as improper dose metering, mouthpiece positioning, or failure to exhale before activation, significantly reduces therapeutic effectiveness. Studies reveal that nearly 50% of patients use DPIs incorrectly, underscoring the need for proper instructions and awareness to ensure optimal drug delivery [86].

## 4. An Overview of Developed Dry Powder Inhaler of Antiviral Agents

A number of antiviral agents have been developed as inhalable dry powder formulation to treat RTIs. These agents are intended to treat specific infection or repurposed agents. Repurposing agents involves the strategy of identifying new therapeutic uses for existing approved or investigational drugs [87]. This approach is often preferred over the development of entirely new drugs for a specific disease due to the lengthy and complex process, as well as the substantial investment and low success rate associated with new drug development [88]. In contrast, repurposing drugs requires less time and investment and generally has a higher success rate [88]. Therefore, the drugs employed for treating RVIs can encompass both antiviral agents and repurposed drugs. A detailed overview of some of the developed inhalable dry powder formulations containing different antiviral agents for RVIs are discussed below.

Remdesivir was developed as inhalable dry powder using thin-film freezing (TFF) technology for treating SARS-CoV-2 infection [89]. The study utilized Captisol, mannitol, lactose, and L-leucine. Captisol was employed to enhance the solubility and stability of remdesivir, while mannitol, lactose, and L-leucine acted as aerosolization enhancers. The resulting formulations exhibited high porosity with a brittle matrix structure, and remdesivir was present in an amorphous form. The dry powder was administered using a Plastiape RS00 inhaler. The optimized formulation, prepared in an acetonitrile/water (50/50) co-solvent system with L-leucine, achieved a fine particle fraction (FPF) of approximately 93%. The dry powder demonstrated physical and chemical stability, and in vitro aerosolization was minimally affected even after one month of storage at 25 °C/60% relative humidity.

Saha et al. [90] developed remdesivir dry powder formulation incorporating disulfiram using spray drying technology and in the presence/absence of leucine with the aim of inhibiting SARS-CoV-2. The researchers chose combinational drugs that have different mechanisms of action. For example, remdesivir inhibits the replication process, and disulfiram inhibits the main protease of SARS-CoV-2. The remdesivir and disulfiram combinational dry powders were within the inhalable size range of 1–5 µm, crystalline and spherical in shape. The remdesivir–disulfiram combination showed >88% ED and >55% FPF when prepared without leucine. The incorporation of leucine significantly enhanced the FPF to more than 60% for the combinations. The prepared dry powders showed similar anti-SARS-CoV-2 activities to the used raw materials, indicating the suitability of the development technique.

Wong et al. [91] developed a carrier-free inhalable favipiravir–theophylline co-crystal dry powder using a spray-drying technique. Favipiravir was chosen for its activity against SARS-CoV-2 and influenza virus, whereas theophylline was chosen for its anti-inflammatory property. The raw materials (favipiravir and theophylline) and developed powder were crystalline in nature. The MMAD and FPF of the optimized formulation were 2.93 μm and 79.3%, respectively, when 5 mg of dry powder was dispersed from a Breezhaler at a flow rate of 60 L/min for 4 s. The developed powder exhibited a porous structure and showed no cytotoxicity on A549 cells even up to a concentration of 1000 μM.

Zhang et al. [92] developed an inhalable dry powder formulation of niclosamide (NCL) using spray–freeze-drying (SFD) technology to enhance pulmonary delivery for potential COVID-19 treatments. The formulation incorporated niclosamide nanocrystals stabilized with Tween-80 and 1,2-distearoyl-sn-glycero-3-phosphocholine (DSPC) and enhanced with leucine to improve dispersibility and flowability. Optimal conditions involved a freezing temperature of −80 °C and a DSPC-to-Tween-80 mass ratio of 2:1, producing spherical microparticles with a fine particle fraction (FPF) of 47.83% and an extra fine particle fraction (eFPF) of 11.80%. The powder achieved a fine particle dose of >3.8 mg when 10 mg of dry powder was inhaled, ensuring efficient deep lung deposition. The addition of leucine contributed to microparticle integrity and reduced moisture absorption, while the low freezing temperature resulted in a porous structure favorable for aerosolization. The formulation maintained stability, and the study highlighted SFD as a scalable method to produce high-dose dry powders for respiratory drug delivery.

Saha et al. [93] developed an inhalable dry powder of ivermectin using spray-drying to target respiratory viral infections, specifically SARS-CoV-2. The formulation included the use of a 23 factorial design with a varying feed concentration (0.2% and 0.8% *w*/*v*), an inlet temperature (80 °C and 100 °C), and the addition of L-leucine (0% and 10% *w*/*w*). The optimized powder (feed concentration: 0.8% *w*/*v*; inlet temperature: 100 °C; 10% *w*/*w* L-leucine) exhibited a fine particle fraction (FPF) of 82.5% and an emitted dose (ED) of 77.9%, ensuring efficient pulmonary deposition. Particle sizes were within the desired 1–5 µm range for effective lung deposition. The addition of L-leucine improved yield and minimized moisture content. The powder demonstrated an amorphous morphology and wrinkle-like particle structure, which are favorable for inhalation. In stability testing, the dry powder maintained consistent physicochemical properties under ambient conditions. Additionally, in vitro assays confirmed that the powder effectively inhibited SARS-CoV-2 replication in Calu-3 cells, with an EC50 comparable to ivermectin in solution, indicating retained antiviral potency.

Panozzo et al. [94] evaluated a dry powder delivery system for laninamivir octanoate (LO) using a Dry Powder Insufflator™ (DPI) in a ferret model to study its effectiveness against influenza A and B infections. The LO formulation consisted of a micronized LO-lactose blend (20:80 *w*/*w*), with LO particles sized between 0.7 and 6.0 µm, enabling pulmonary deposition.

Aziz et al. [95] developed a dry powder formulation of oseltamivir phosphate (OP) for inhalation therapy targeting viral pneumonia, including influenza and COVID-19. The formulation utilized micronized OP blended with various excipients, including trehalose, lactose, glucose, and mannitol, in ratios of 1:1, 1:3, and 3:1. Trehalose emerged as the optimal excipient due to its superior aerosolization performance and ability to enhance fine particle dose (FPD). Micronized trehalose formulations achieved fine particle fractions (FPF) up to 62.5%, delivering an FPD of 6.2 mg using the UNI-Haler inhaler. Trehalose acted as a spacer within cohesive OP particle meshes, promoting efficient deagglomeration upon inhalation. Stability testing confirmed the integrity of trehalose’s crystalline structure and its role in facilitating particle dispersion. The powder demonstrated no cytotoxicity on Calu-3 lung epithelial cells, indicating safety for pulmonary administration. This study highlights the potential of high-dose inhalable OP formulations for targeted respiratory therapy using DPIs.

Seow et al. [96] developed a dual-targeting dry powder formulation of tamibarotene using spray–freeze-drying (SFD) and spray-drying (SD) to deliver antiviral agents to both the upper and lower respiratory tracts via intranasal administration. The formulations incorporated tamibarotene and 2-hydroxypropyl-β-cyclodextrin (HPBCD) as an excipient to enhance solubility, with ultrasonic and two-fluid nozzles employed to create particles >10 µm for nasal deposition and <5 µm for pulmonary deposition. The powders exhibited a bimodal size distribution and customizable deposition profiles, with the nasal fraction (NF) and fine particle fraction (FPF) ratios adjusted by varying the blending ratio of particle sizes. SD formulations demonstrated better NF:FPF ratios and were less influenced by flow rate, making them more suitable for scalable manufacturing. The formulations showed spherical morphology, amorphous characteristics, and rapid dissolution profiles, with improved solubility over unformulated tamibarotene. This approach demonstrates the potential for dual-targeting powder aerosols to treat respiratory viral infections, including SARS-CoV-2 and influenza, using portable nasal devices.

Leung et al. [97] developed a dry powder delivery system for zanamivir (Relenza^®^) to treat influenza in intubated patients, addressing the risks of using nebulized solutions, which can obstruct ventilator filters. The system incorporated a manual ventilation bag and a delivery chamber to aerosolize the powder through tracheostomy or endotracheal tubes. Two inhalers, Diskhaler^®^ and Osmohaler™, were evaluated for performance. The Osmohaler™ demonstrated superior efficiency, delivering a dose of 30.5% and achieving a fine particle fraction (FPF) of 14.5%, compared to the Diskhaler^®^ (delivered dose of 18.1% and FPF of 3.4%). Larger endotracheal tubes (9.0 mm, internal diameter) improved FPF (20.4%) without significantly affecting the delivered dose.

## 5. An Overview of Marketed Dry Powder Inhaler Containing Antiviral Agents

The market for DPIs is very hopeful as the market of this sector had a value of over USD 835 million in 2020 [98]. It is expected to have a compound annual growth rate of >5% from 2021 to 2026 [98]. This growth can be attributed to the rising occurrence of respiratory diseases, which presents new opportunities and drives the demand for various DPIs. As a result, the industrial growth rate is expected to increase. Currently, different DPI devices are available in the market for different respiratory diseases. Some examples are Foradil Aerolizer (formoterol fumarate), Adavir Diskus (fluticasone propionate and salmeterol xinafoate), and Serevent Diskus (salmeterol xinafoate) for COPD; Budelin Novolizer (Budesonide), Salbulin Novolizer (salbutamol), and Asmasal Clickhaler (salbutamol sulfate) for asthma; and Tudorza Pressair (aclidinium bromide) for bronchospasm [99]. So, it can be easily understood that the existing marketed DPIs are mostly for bronchodilators. However, the number of DPIs for RVIs is very limited. Currently, two antivirals, namely zanamivir and laninamivir, are available in the market as DPI formulations to treat RVIs.

Zanamivir and laninamivir belong to the class of neuraminidase inhibitors and are mainly used against both influenza A and B strains [100,101,102]. The zanamivir DPI (Brand name: Relenza^®^) is developed by GalxoSmithKline, London, UK, and delivered using a Diskhaler (ROTADISK) (Table 2). Every ROTADISK has four double foil blisters containing a powder mixture of zanamivir (5 mg) and lactose monohydrate (20 mg). Lactose monohydrate is used as an excipient to ensure better flowability of the drug. The shelf life of Relenza is 10 years and should be stored at ≥30 °C. Zanamivir is delivered as a DPI due to its poor oral bioavailability (<17%). Relenza is applicable for both children (≥5 years) and adult patients. The recommended dose of Relenza is two inhalations (2 × 5 mg) twice daily for 5 days, providing a total daily inhaled dose of 20 mg. To ensure the maximum therapeutic activity, the treatment should start within two days after the onset of symptoms. Apparently, 15% of the powder can reach the lower respiratory tract, and the remaining portions deposit in the oropharynx and last up to 24 h. Inhaled zanamivir was found to be more effective compared to oral oseltamivir in reducing the symptom severity of influenza-infected patients. Respiratory distress, cough, and bronchospasm in asthmatic patients are some of the reported adverse effect of zanamivir.

The laninamivir DPI is developed by Daiichi Sankyo, Tokyo, Japan (Brand name: Inavir^®^) and only approved in Japan (Table 2). Laninamivir is exhibited as laninamivir octanoate. Like Relenza, Inavir also contains lactose in its formulation. The recommended dose is 40 mg of laninamivir octanoate (a single inhaled dose) for adults and children over 10 years of age. For children below 10 years of age, the recommended dose is 20 mg of laninamivir octanoate (a single inhaled dose). Inavir is well tolerated by patients, except for a few reported adverse effects of nausea and vomiting.

Based on the aforementioned scenario, it becomes evident that the current market for antivirals containing DPIs for RVIs is quite limited, despite the promising laboratory-based formulations of dry powder antivirals. Consequently, it is crucial to prioritize efforts in transitioning these promising antimicrobial DPIs from the experimental stage to practical application, ensuring their availability and effectiveness in clinical settings.

## 6. Inhaled Combinational Formulations: An Area to Explore

The available marketed and published inhalable dry powders of antivirals mostly contain single agents, and the reported inhalable dry powders of combinational antivirals are very limited. However, for viral infections, drug combinations have been found to be more effective [103]. Wisely selected combinational drugs can offer various advantages over single agents. One primary benefit is the synergistic effect achieved when combining multiple antiviral agents, which can enhance therapeutic efficacy and improve patient outcomes [104]. Synergistic drug combinations enable the simultaneous targeting of multiple viral mechanisms, potentially leading to better viral inhibition than single-drug approaches [105]. A list of different antiviral combinations showing synergistic activity against different respiratory viruses is presented in Table 3.

Another significant advantage of combinational therapies is their role in reducing the risk of drug resistance [106]. Monotherapies can often lead to resistance as viruses mutate to evade single-agent mechanisms [106]. By attacking multiple viral pathways simultaneously, combinational therapies make it more challenging for viruses to develop resistance, as they would need to adapt to several mechanisms at once. This approach has been effectively demonstrated in HIV therapy, where combining antiviral agents has significantly reduced the development of resistant strains, and similar strategies are being explored for influenza and RSV [107]. RVIs often occur alongside secondary viral or bacterial infections, complicating treatment and leading to more severe clinical outcomes [108,109]. In cases of co-infection with respiratory viruses, a combination of antiviral agents allows for broad-spectrum treatment that can effectively target multiple pathogens simultaneously. For example, combining ribavirin with a broad-spectrum antiviral could be beneficial in patients co-infected with RSV and influenza, providing comprehensive antiviral coverage, potentially reducing the severity and duration of illness. Moreover, inhaled combinational therapies can improve patient compliance by simplifying treatment regimens, a crucial aspect for patients with chronic respiratory illnesses or those at risk for frequent infections. Instead of managing multiple oral or intravenous medications, patients can receive a single inhaled dose, which is often more convenient and less invasive. This convenience not only enhances adherence but also improves treatment efficacy by ensuring that patients consistently receive their full regimen, as has been observed in chronic obstructive pulmonary disease (COPD) patients with respiratory infections.

**Table 3:** List of synergistic drug combinations tested against different respiratory viral pathogens.

Drug A	Mode of Action	Drug B	Mode of Action	Pathogens	Cell Line Used	Ref
Nitazoxanide	Replication inhibitor	Oseltamivir	Neuraminidase inhibitor	Influenza virus	MDCK cell line	[110]
Nitazoxanide	Replication inhibitor	Zanamivir	Neuraminidase inhibitor	Influenza virus	MDCK cell line	[110]
Oseltamivir	RdRp inhibitor	Rimantadine	Viral replication	Influenza virus	MDCK cell line	[111]
Oseltamivir	RdRp inhibitor	Zanamivir	Neuraminidase inhibitor	Influenza virus	MDCK cell line	[112]
Oseltamivir	Neuraminidase inhibitor	Favipiravir	RdRp inhibitor	Influenza virus	MDCK cell line	[113]
Remdesivir	RdRp inhibitor	Ebselen	Protease inhibitor	SARS-CoV-2	Vero cell line	[114]
Remdesivir	RdRp inhibitor	Disulfiram	Protease inhibitor	SARS-CoV-2	Vero cell line	[114]
Remdesivir	RdRp inhibitor	Ivermectin	Importin α/β1 inhibitor	SARS-CoV-2	264.7 murine macrophage cell line	[115]
Remdesivir	RdRp inhibitor	Nitazoxanide	Entry inhibitor	SARS-CoV-2	Vero E6 cell line	[116]
Nitazoxanide	Entry inhibitor	Umifenovir	Entry inhibitor	SARS-CoV-2	Vero E6 cell line	[116]
Nitazoxanide	Entry inhibitor	Emetine dihydrochloride hydrate	Replication inhibitor	SARS CoV-2	Vero E6 cell line	[116]
Nitazoxanide	Entry inhibitor	Amodiaquine	Entry inhibitor	SARS-CoV-2	Vero E6 cell line	[116]
Favipiravir	RdRp inhibitor	Ivermectin	Importin α/β1 inhibitor	SARS-CoV-2	Vero E6 cell line	[117]
Otamixaban	Entry inhibitor	Camostat	TMPRSS2 inhibitor	SARS-CoV-2	Vero E6 cell line	[118]
Otamixaban	Entry inhibitor	Nafamostat	TMPRSS2 inhibitor	SARS-CoV-2	Vero E6 cell line	[118]
Remdesivir	RdRp inhibitor	Brequinar	Replication inhibitor	SARS-CoV-2	Vero E6 cell line	[119]
Molnupiravir	RdRp inhibitor	Brequinar	Replication inhibitor	SARS-CoV-2	Vero E6 cell line	[119]
Cepharanthine	Entry inhibitor	Nelfinavir	Replication inhibitor	SARS-CoV-2	Vero E6 cell line	[120]
Lumicitabine (ALS8176)	RdRp inhibitor	RSV604	RdRp inhibitor	RSV	Hep-2 cells	[121]
Lumicitabine (ALS8176)	RdRp inhibitor	BMS433771	Inhibits fusion protein	RSV	Hep-2 cells	[121]
Presatovir (GS5806)	Inhibits fusion protein	BMS433771	Inhibits fusion protein	RSV	Hep-2 cells	[121]
Lumicitabine (ALS8176)	RdRp inhibitor	Ziresovir	Inhibits fusion protein	RSV	Hep-2 cells	[121]
Lumicitabine (ALS8176)	RdRp inhibitor	Presatovir (GS5806)	Inhibits fusion protein	RSV	Hep-2 cells	[121]
Glycyrrhizic acid	Inhibits entry	Ephedrine	Inhibits entry	RSV	A549 cells	[122]
Fluticasone propionate	Inhibits VEGF, FGF-2 production	Salmeterol	Inhibits VEGF, FGF-2 production	Rhinovirus	Bronchial epithelial cells	[123]
Rupintrivir	Inhibits replication by targeting 3C protease	Itraconazole	Targets oxysterol-binding protein and inhibits replication	Enterovirus 71	RD cells	[124]
Rupintrivir	Inhibits replication by targeting 3C protease	Favipiravir	Inhibits replication targeting RNA polymerase	Enterovirus 71	RD cells	[124]
Suramin	Prevents attaching to the host cell surface receptors	Favipiravir	Inhibits replication targeting RNA polymerase	Enterovirus 71	RD cells	[124]
Rupintrivir	Inhibits replication by targeting 3C protease	Vemurafenib	Inhibits replication	Enterovirus 1	A549 cells	[125]
Rupintrivir	Inhibits replication by targeting 3C protease	Pleconaril	Inhibits replication by binding to viral capsid	Enterovirus 1	A549 cells	[125]
Pleconaril	Inhibits replication by binding to viral capsid	Vemurafenib	Inhibits replication	Enterovirus 1	A549 cells	[125]
Rupintrivir	Inhibits replication by targeting 3C protease	Interferon	Inhibits replication	Enterovirus 71	Vero cells	[126]
Ribavirin	RdRp inhibitor	Gemcitabine	Inhibits replication	Enterovirus 71	Vero cells	[127]
Rupintrivir	Inhibits replication by targeting 3C protease	Cycloheximide	Inhibits replication	Enterovirus 71	A549 cells	[125]

## 7. Conclusions

Inhalable dry powders have emerged as a promising treatment approach for treating respiratory viral infections. DPIs offer targeted delivery, efficient drug distribution, ease of use, portability, and better product stability. However, the number of marketed DPIs for respiratory viral infections is very few. As a result, millions of people are dying each year to these respiratory diseases. To address this critical gap, researchers from pharmaceutical industries and academia should work collaboratively and intensively to bring promising dry powder formulations from the bench to the bedside. This will not only ensure enhanced patient compliance but also help us to fight against future viral epidemics or pandemics. Besides that, especial attention should be given to develop combinational inhalable dry powders that have additional advantages over single-agent inhalable dry powders. Inhaled combination dry powder formulations offer improved therapeutic efficacy by targeting multiple pathways simultaneously, enhancing drug synergy while reducing the required dose and potential side effects compared to single-drug inhalers. They also improve patient adherence by simplifying treatment regimens.

## Figures and Tables

**Table 1:** Simple comparison between dry powder inhaler, pressurized metered-dose inhaler, and nebulizer.

	Dry Powder Inhaler	Pressurized Metered Dose Inhaler	Nebulizer
Medication type	Powder	Liquid	Liquid
Physical and chemical stability	More	Less than powder	Less than powder
Activation	Breath-activated	Pressurized canister	Through compressed gas/electricity
Inhalation and device coordination	Required	Required	Not required
Portability	Highly portable	Highly portable	Less portable and requires set up
Maintenance	Minimal	Requires cleaning of inhaler and canister	Requires cleaning of nebulizers and replacement of nebulizer parts if needed
High-dose delivery	Yes	No	Yes
Transmission	No/Less	No/Less	High

**Table 2:** Marketed dry powder inhalers for respiratory tract infections.

Product	Active Ingredient	Reported Excipients	Indication	Dose per Day	Manufacturer
Relenza Diskhaler	Zanamivir	Lactose monohydrate	Influenza	5 mg/twice per day	Galxo SmithKline, UK
Inavir	Laninamivir octanoate	Lactose monohydrate	Influenza	40 mg/once a day	Daiichi Sankyo, Japan

## Data Availability

Not applicable.
